# 3D hydrogel breast cancer models for studying the effects of hypoxia on epithelial to mesenchymal transition

**DOI:** 10.18632/oncotarget.25891

**Published:** 2018-08-14

**Authors:** Ying Wang, Sameer Mirza, Shaohua Wu, Jiping Zeng, Wen Shi, Hamid Band, Vimla Band, Bin Duan

**Affiliations:** ^1^ Mary & Dick Holland Regenerative Medicine Program, University of Nebraska Medical Center, Omaha, NE, USA; ^2^ Department of Biochemistry and Molecular Biology, School of Basic Medical Sciences, Shandong University, Jinan, China; ^3^ Department of Genetics, Cell Biology and Anatomy University of Nebraska Medical Center, Omaha, NE, USA; ^4^ Eppley Institute for Research in Cancer and Allied Diseases, University of Nebraska Medical Center, Omaha, NE, USA; ^5^ Fred & Pamela Buffett Cancer Center, University of Nebraska Medical Center, Omaha, NE, USA; ^6^ Division of Cardiology, Department of Internal Medicine, University of Nebraska Medical Center, Omaha, NE, USA; ^7^ Department of Surgery, College of Medicine, University of Nebraska Medical Center, Omaha, NE, USA; ^8^ Department of Mechanical and Materials Engineering, University of Nebraska-Lincoln, Lincoln, NE, USA

**Keywords:** lysyl oxidase, epithelial-mesenchymal transition, hydrogel, hypoxia

## Abstract

Solid tumors are 3D assemblies of cancer cells, together with multiple stromal cell types within an extracellular matrix. Yet, the vast majority of cell-based studies to characterize oncogenesis and discovery of new anti-cancer drugs is conducted using conventional 2D monolayer culture systems, where cells are grown on plastic substratum under normoxic environments. In current study, we generated 3D breast cancer cell culture platform consists of photocrosslinkable hydrogels and encapsulated isogenic primary (21PT) and a metastatic (21MT-2) breast cancer cell lines derived from the primary tumor and pleural effusion from the same patient. We demonstrated that hypoxia decreased cellular assembly size and density, and promoted epithelial to mesenchymal transition (EMT) process, without affecting cell viability. Next, we showed hypoxia enhanced breast cancer cell migration, and expression and secretion of lysyl oxidase (LOX), which is copper-dependent amine oxidase and has the primary function to drive the crosslinking of collagen and elastin and is regulated by hypoxia. Furthermore, to recapitulate *in vivo* situation, we generated breast cancer and lung cells (derived from the same patient) contact model by stacking 3D hydrogel constructs with breast cancer cells onto lung mesenchymal cells (LMC) laden-hydrogel and then showed breast cancer cells migrated towards LMC during hypoxia. Lastly, as a validation of this model for future screen of therapeutic agents, we demonstrated that LOX inhibitor exhibited a significant decrease in breast cancer cell viability, migration, and EMT. Taken together, these results validate the use of hydrogels based models to examine hypoxia related EMT in breast cancer cells.

## INTRODUCTION

Breast cancer proliferation, progression, and metastasis are highly influenced by the physical and cellular microenvironments [1, 2]. Conventional two-dimensional (2D) monolayer culture systems are routinely used in the pharmaceutical industry to develop therapeutic agents [3, 4]. However, in 2D tumor models, cancer cells sit on a flat surface with almost half of the cells’ surface directly in contact with plastic substrate and thus lack native cell-cell contact, and cell-matrix interactions [5, 6]. More importantly, cancer cells grown on the 2D stiff plastic surface are known to exhibit different cytokine secretion capacity, cell behaviors and response to anti-cancer drugs. For example, IL-8 expression was constitutively increased in human oral squamous cell carcinoma in three-dimensional (3D) environments but not in 2D monolayers [7], and MCF-7 cancer cells grown in the 3D model had reduced sensitivity to doxorubicin in comparison with cells cultured in 2D condition [8]. Not surprisingly, several agents screened by 2D culture failed in the *in vivo* settings and many promising compounds may not reach to the clinical trials due to lack of proper microenvironment for cancer cells [9, 10]. These findings underscore the need for 3D culture models with proper extracellular matrix (ECM) like environment and cell-cell interactions to recapitulate the breast cancer microenvironment and to bridge the gap between monolayer cultures and animal model studies, which not always predict similar therapeutic outcome. To this end, many techniques and materials have been used to engineer 3D breast cancer models, including material-free cancer spheroids [11, 12], scaffold based matrix [13, 14], microfluidic devices [15, 16], 3D bioprinting [17, 18], and assembly techniques [19, 20]. Hydrogel based cancer models have similar stiffness to the native adipose tissues and many natural hydrogels (like hyaluronic acid-HA, collagen, and fibrin) are obtained from the ECM [21–23]. More importantly, hydrogel based cancer models are amenable to control stiffness, structure, size, and various components.

Along with the cell-cell and cell-matrix interaction in the microenvironment, hypoxia is one of the most important determinant of cancer cell behavior [24]. In fact, intratumoral hypoxia is a common event in breast cancer progression and it correlates with poor outcome [25]. It has been demonstrated that cellular responses to hypoxic environment are primarily regulated by hypoxia-inducible factors (HIF) [26, 27] and HIF activates numerous pathways that promote primary tumor vascularization and proliferation [28], stromal cell recruitment [29], and extravasation at sites of metastasis [30]. Investigators have demonstrated that hypoxia was observed only in the dense 3D breast cancer cell spheroids and played an important role in drug resistance [5]. Similarly, other investigators have reported that hypoxia induced changes in gene expression of breast cancer cells varied greatly based on its 2D or 3D culture environment, and genes regulated by dimensionality also depended on oxygen tension [7]. Surprisingly, very few studies have established versatile 3D systems where hypoxia signaling directly links to pro-metastatic traits, such as EMT.

Hypoxia also regulates pre-metastatic niche formation by altering ECM deposition and remodeling [31], mediating microvesicle formation and release [32], and controlling various cytokine (or other secretomes) secretion [33] to prime the target organ and provide an initial site for tumor cell colonization. Hypoxia can induce the secretion of lysyl oxidase (LOX), which is copper-dependent amine oxidase and has the primary function to drive the crosslinking of collagen and elastin [34, 35]. Importantly, several studies have demonstrated that LOX is associated with breast cancer bone metastasis [36–38]. LOX is also known to be critical for pre-metastatic niche formation by crosslinking collagen IV in the basement membrane and CD11b+ myeloid cell recruitment [39]. In addition, LOX supports the attachment and survival of cancer cells to and in the bone matrix and dissemination in the bone marrow [40, 41]. However, it is still unclear whether hypoxia induced LOX is critical for breast cancer lung metastasis and how LOX inhibition affects pre-metastatic niche formation in the lung tissue.

In the present study, we first generated 3D breast cancer cell culture platform consisting of photocrosslinkable methacrylated hyaluronic acid (Me-HA) and methacrylated gelatin (Me-Gel). We used two isogenic cell lines from one patient, one primary (21PT) and one metastatic (21MT-2) which were obtained from primary and lung metastasis of the same patient, respectively and thus provide an excellent model to validate the system. Our systematic investigation of effect of hypoxia on breast cancer cell assembly and gene expression within 3D culture showed hypoxia enhanced EMT, increased LOX expression and activity, and migration onto lung mesenchymal cells (LMC, derived from the same patient) laden hydrogel. Under these conditions, we observed LOX inhibitors decreased cancer cell viability, migration and EMT behavior. Taken together, we have generated 3D breast cancer cell hydrogel models under hypoxia, which faithfully mimic the *in vivo* breast tumor environment, and will therefore provide a more accurate model to identify novel breast cancer therapies targeted at the hypoxia response pathway.

## RESULTS

### Effects of hypoxic environment on cell viability and self-assembly

**Figure 1:**
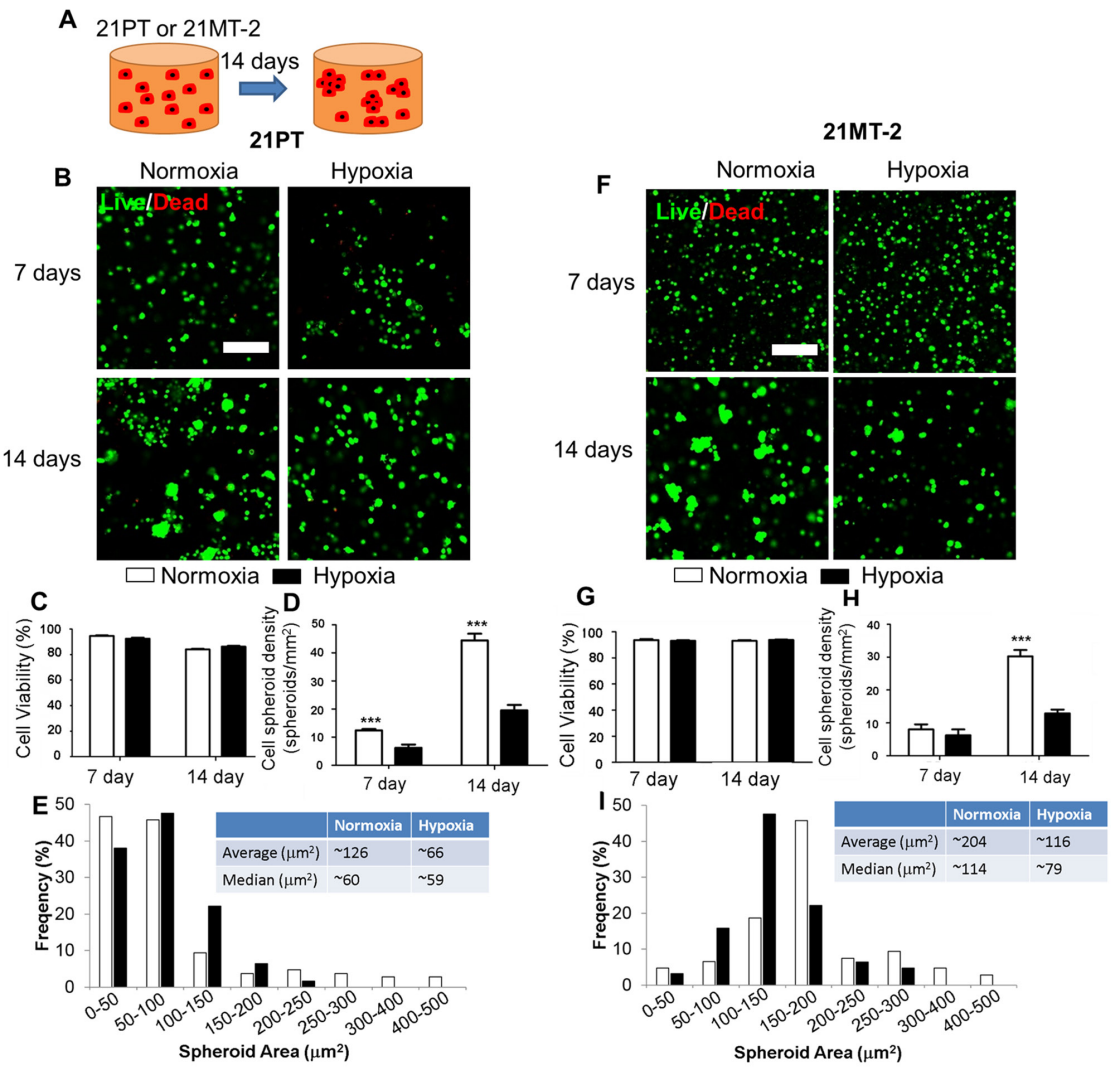
Effects of hypoxia on viability and self-assembly of 21PT and 21MT-2 within hydrogel constructs **(A)** Schematic of experimental design with homogeneous cell encapsulation within Me-HA/Me-Gel hydrogels. The cell-hydrogel constructs were maintained in normoxia or hypoxia for 14 days and had spontaneous self-assembly into breast cancer/tumor spheroid like structure; **(B, F)** Representative IF images of living cells (green) and dead cells (red) within hydrogels after 14-day culture (scale bars = 200 μm); Cell viability **(C, G)**, spheroid density **(D, H)**, and spheroid area distribution **(E, I)** analysis of breast cancer cells encapsulated within the hydrogels. (B, C, D, E) 21PT, (F, G, H, I) 21MT-2; ^***^p<0.001.

Me-HA/Me-Gel hydrogels were fabricated by photocrosslinking. Scanning electron microscopy (SEM) images showed that the microstructure morphologies of Me-HA hydrogels exhibited irregular pore shapes and relatively open 3D network structure (Supplementary Figure 1). The stiffness of crosslinked Me-HA/Me-Gel hydrogels was measured to be 1.16±0.96 kPa, which is comparable to native adipose tissue (∼2 kPa) [21]. 21PT or 21MT-2 cells were encapsulated within Me-HA/Me-Gel hydrogels and most of the cells survived the encapsulation process and kept alive throughout 14-day culture in both nomoxia and hypoxia (Figures 1B and 1F). Although the cell viability for 21PT slightly decreased after 14-day culture (Figure 1C), both cell types had high cell viability (>80%) and there was no significant difference between normoxic and hypoxic environment (Figures 1C and 1G). Noticeably, both 21PT and 21MT-2 cells tended to self-assemble into spheroid or cluster like structure (Figures 1B and 1F). With increasing the culture time, the size of spheroid or the cluster structure significantly increased (Figures 1D and 1H). In the normoxic environment, the cancer spheroids had significantly higher density (Figures 1D and 1H) and had larger, average and median size and broader distribution comparing to those in hypoxic environment (Figures 1E and 1I).

### Hypoxic environment promoted epithelial-mesenchymal (EMT) transition

**Figure 2:**
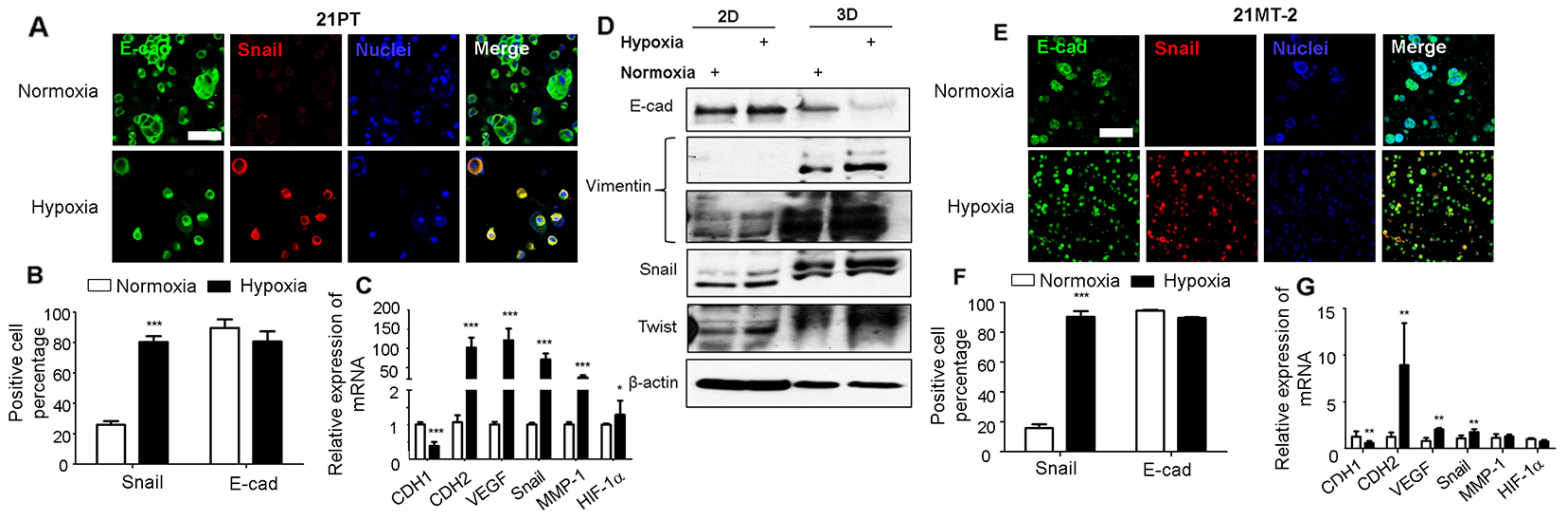
Effects of hypoxia on EMT of 21PT and 21MT-2 within hydrogel constructs **(A, E)** Representative IF staining for E-cadherin (E-cad, green), Snail (red) and nuclei (blue) of breast cancer cells within hydrogel scaffolds in normoxia or hypoxia after 14-day culture (scale bars = 100 μm); **(B, F)** Percentage of cells that were stained positive to E-cad and Snail based on IF staining analysis; **(C, G)** qPCR analysis of CDH1, CDH2, VEGF, Snai1, MMP1 and HIF-1α expression of breast cancer cells within hydrogels. Relative gene expression is presented as normalized to actin and expressed relative to breast cancer cells within hydrogels in normoxic environment (n=3). (A, B, C) 21PT, (E, F, G) 21MT-2; ^*^p<0.05, ^**^p<0.01, ^***^p<0.001; **(D)** Western blotting was performed to compare the protein expressions of 21PT cells conditioned in normoxia or hypoxia in 2D and 3D culture with indicated antibodies and βactin was used as a loading control..

Immunofluorescent (IF) staining showed that in the normoxic environment, 21PT assembled into spheroid like structure with very limited Snai1 expression, whereas in the hypoxic environment, the Snai1 expression was clearly observed (Figure 2A). The Snai1 positive cell density was much more in hypoxia than in normoxic environment, and the E-cadherin positive cell density was lower (Figure 2B). QPCR results also showed that the hypoxia significantly downregulated CDH1 expression, which is a critical gene for EMT, and additionally significantly upregulated CDH2, VEGF, Snai1, MMP-1 and HIF 1α, which are known to be highly associated with EMT process (Figure 2C). We further conducted western blot analysis and compared several EMT related protein expression in 21PT cells conditioned in normoxia or hypoxia in 2D and 3D culture. Figure 2D showed that comparing to 2D culture 21PT cells expressed less E-cad, and more Vimentin, Snail, and Twist in 3D culture, indicating an enhanced EMT phenotypes. In addition, hypoxic condition decreased expression of E-cad, and promoted the expression of Vimentin, Snail, and Twist, especially in the 3D culture. These results are consistent with qPRC and IF staining results. Taken together, these results indicated that in this 3D hydrogel model hypoxia promoted EMT process of 21PT cells. Similarly, hypoxia treatment increased Snail positive cells in 21MT-2 laden hydrogels (Figures 2E and 2F). The expression of CDH1 was downregulated, while the expression of CDH2, VEGF, and Snai1 were significantly upregulated in the hypoxic environment (Figure 2G). Surprisingly, hypoxia did not have obvious effects on HIF1α expression in 21MT-2, a metastatic cell line, a clear difference from 21PT cells.

### Effects of hypoxia on LOX expression and secretion

**Figure 3:**
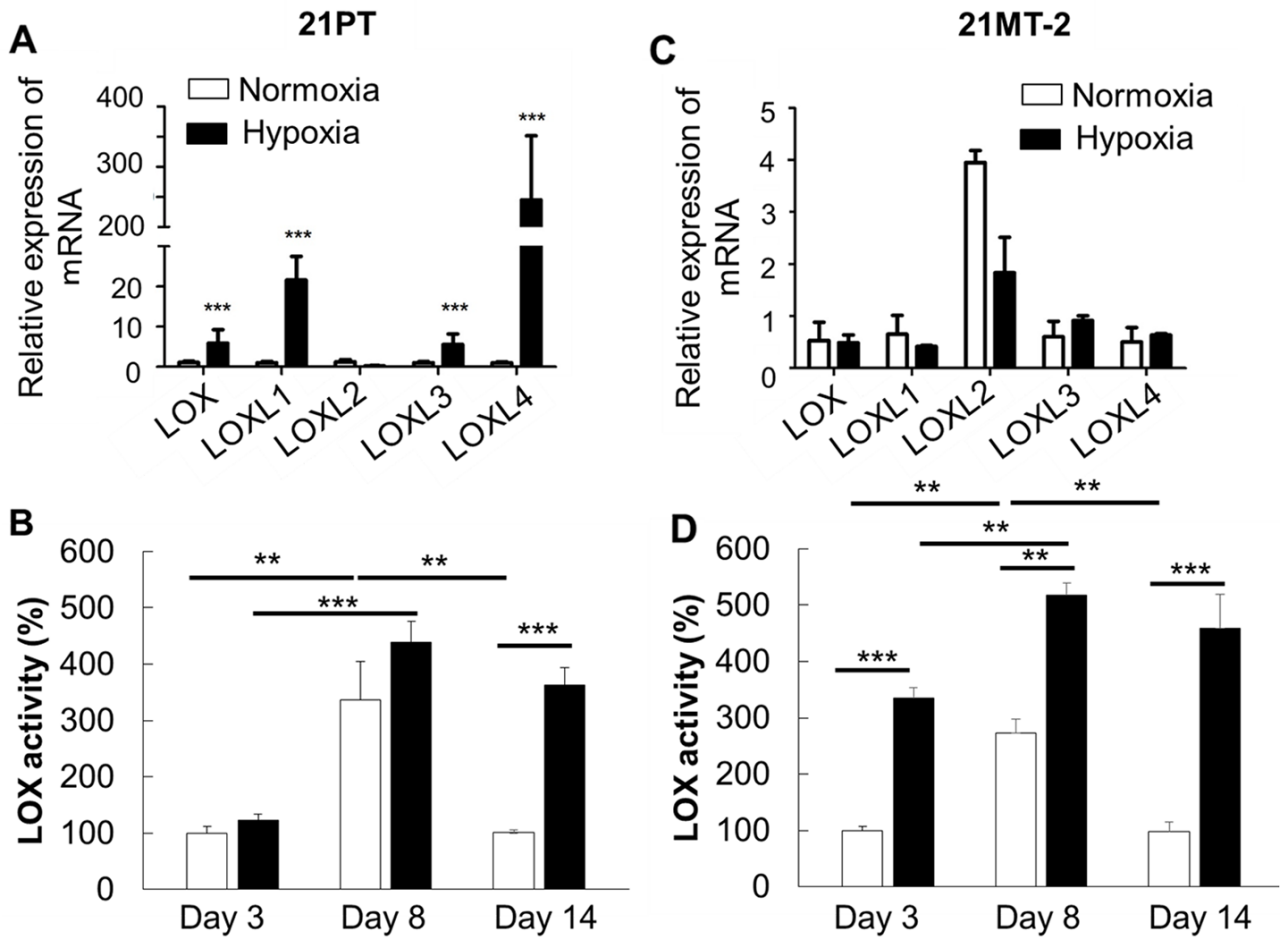
Effects of hypoxia on LOX and LOX like enzymes expression and secretion **(A, C)** qPCR analysis of LOX, LOXL1, LOXL2, LOXL3, and LOXL3 expression of breast cancer cells within hydrogels. Relative gene expression is presented as normalized to actin and expressed relative to breast cancer cells within hydrogels in normoxic environment (n=3); **(B, D)** LOX secretion in the media at different time points (n=5). Data are normalized to the average of 21PT or 21MT2 group in normoxia at day 3. (A, C) 21PT, (B, D) 21MT-2; ^**^p<0.01, ^***^p<0.001.

LOX and its family members LOX-like proteins (LOXL) 1-4 are known to be regulated at both transcriptional and post-transcriptional levels, and have both intracellular and extracellular functions [42, 43]. Therefore, we evaluated the LOX and LOXL1-4 expression in the 21PT or 21MT-2 laden hydrogels, as well as determined the secreted LOX activity in the culture media. As shown in Figure 3A, after 14 day culture, hypoxic environment significantly promoted lox family expression just except for LOXL2. At day 3 and 8, the LOX activity in 21PT laden hydrogel constructs was comparable in both normoxia and hypoxia (Figure 3B). At day 14, the secreted LOX showed significantly higher activity in the hypoxic group. Surprisingly, in 21MT-2 cells, LOX gene expression were similar between normoxia and hypoxia conditions (Figure 3C), but hypoxia significantly promoted LOX secretion (Figure 3D). In addition, the secreted LOX activity increased first and then decreased with culture time. This probably relates to the cancer cell migration, and self-assembly status of cells. These results indicate that the LOX expression and secretion are temporal and spatial, and may depend on the cancer stage, primary vs. metastatic.

### 21PT cells exhibited higher EMT gene expression and migration capacity under hypoxia condition

**Figure 4:**
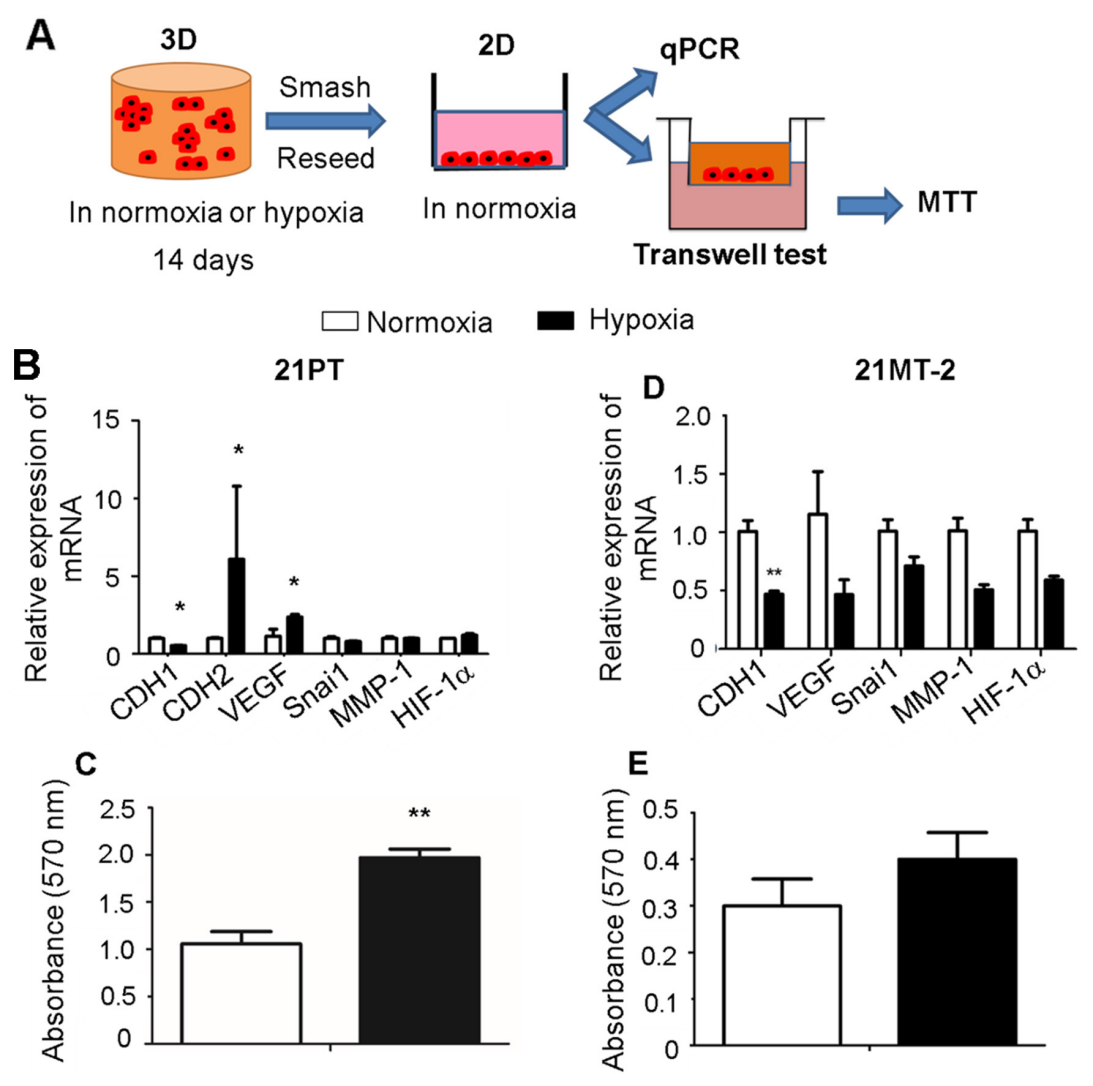
Effects of hypoxia on breast cancer cell migration in response to hypoxic and normoxic pre-conditioning are stage dependent **(A)** Schematic of experimental design. The cell-hydrogel constructs were maintained in normoxia or hypoxia for 14 days and then smashed to release the encapsulated cells. The released cells were reseeded onto the 2D 6-well culture plates for another 3 days in normoxia and then collected for either qPCR assay or transwell/Boyden chamber assay with further MTT assay determine the migrated cell metabolism. **(B, D)** qPCR analysis of CDH1, CDH2, VEGF, Snai1, MMP1 and HIF-1α expression of migrated breast cancer cells. Relative gene expression is presented as normalized to actin and expressed relative to the migrated breast cancer cells that pre-conditioned in normoxic environment (n=3); **(C, E)** MTT assay for the migrated breast cancer cells. (B, C) 21PT, (D, E) 21MT-2; ^*^p<0.05, ^**^p<0.01.

We next evaluated how hypoxia affected 21PT and 21MT-2 migration capacity after cells were released from the hydrogel. The 3D cell laden hydrogel constructs were conditioned in either normoxic or hypoxic environment for 14 days and then were smashed to release from the hydrogels. The released cells were reseeded onto the 2D culture plates for another 3 days in normoxia and then collected for either qPCR assay or transwell/Boyden chamber assay (Figure 4A). For 21PT, the qPCR results showed that 21PT in previous hypoxic condition expressed much less CDH1, and much more CDH2 and VEGF (Figure 4B). In addition, more cells migrated from upper chamber to lower chamber in the transwell assay, as shown in MTT results in Figure 4C. Taken together, these results indicate hypoxic environment promotes EMT process and enhances migration capacity of cells. However, for 21MT-2, only downregulation of CDH1 was observed under hypoxic condition (Figure 4D). There was also no difference for MTT assay between previous normoxic and hypoxic treatment (Figure 4E). These results suggest an inherent difference between two cell lines or difference in primary vs metastatic cells.

### Hypoxia promoted 21PT migration to LMC populated constructs

**Figure 5:**
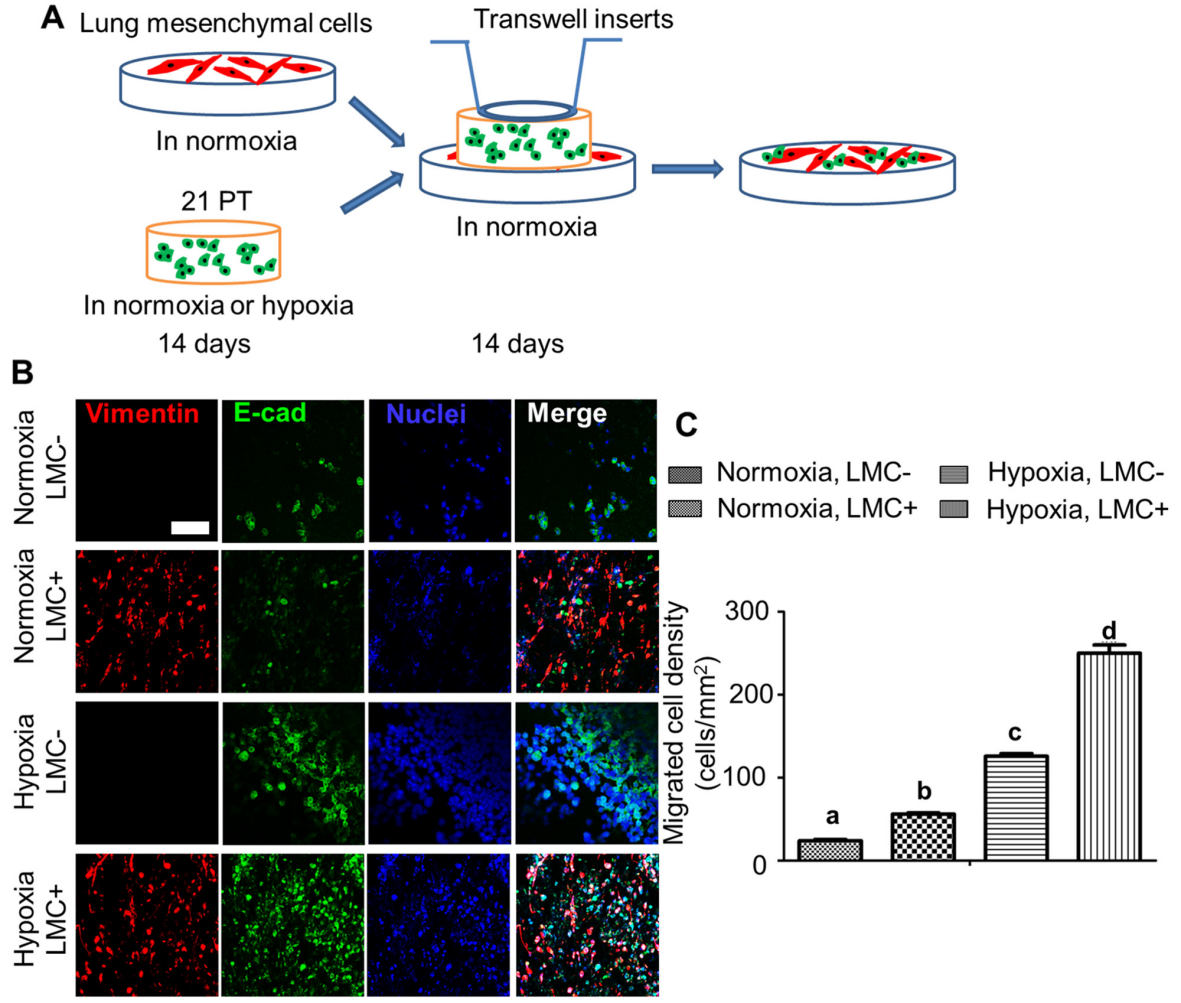
Hypoxia and LMC promoted 21PT migration from the breast cancer cell laden constructs to the hydrogel matrix **(A)** Schematic of breast cancer lung metastasis/migration model. LMC were seeded onto Me-Gel hydrogel disc and conditioned in normoxia for 14 days. During the same time, 21PT laden hydrogel constructs were cultured in either normoxia or hypoxia for 14 days. Then the 21PT laden hydrogel constructs were stacked onto the Me-Gel hydrogel discs with and without LMC with a transwell chamber on the top to make sure the hydrogel discs were contacted. The whole constructs were conditioned in LMC medium in normoxic environment for another 14 days; **(B)** Representative IF staining for vimentin (red), E-cad (green) and nuclei (blue) for migrated 21PT and LMC after 14-day culture (scale bars = 100 μm); **(C)** Semi-quantitative measurement of migrated 21PT density based on counting the E-cad positive cells (n=5; bars that do not share letters are significantly different from each other, p<0.05).

Availability of LMC from the same patient provided a great opportunity to generate a contact model by stacking 21PT laden hydrogels onto LMC laden constructs (Figure 5A). Figure 5B showed that no matter 21PT laden hydrogels were previously conditioned in normoxic or hypoxic environment, more 21PT migrated and attached to LMC seeded hydrogels in comparison with control hydrogels after 14-day co-culture. In addition, 21PT exhibited more migration tendency upon hypoxic treatment when cultured with LMC and upon hypoxic treatment (Figure 5C).

### LOX inhibitor did not affect 21PT cell viability, but inhibited their migration capacity

**Figure 6:**
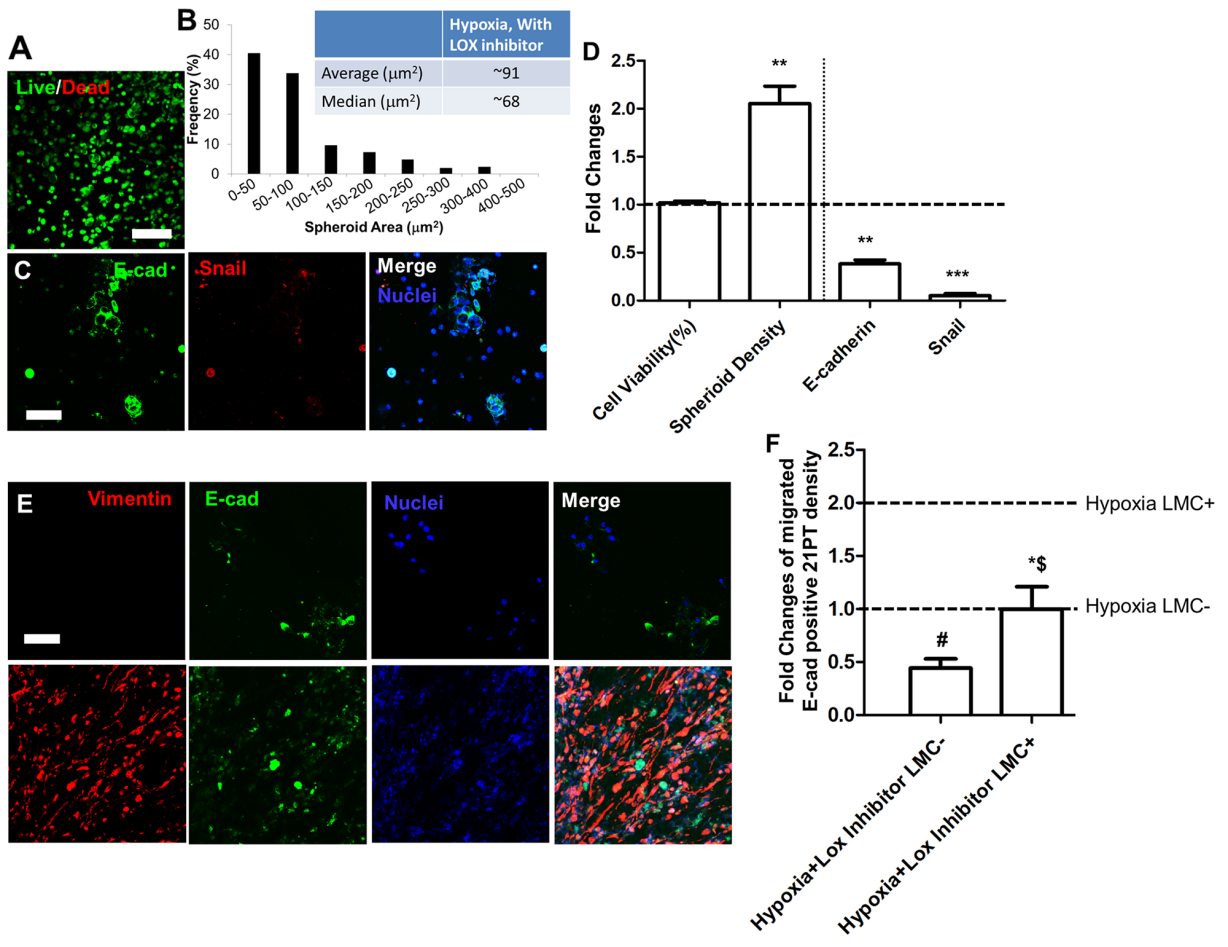
Effects of LOX inhibitor on 21PT viability, EMT, and migration **(A)** Representative live/dead image of 21PT within hydrogels with addition of LOX inhibitor (scale bars = 200 μm); **(B)** Cell spheroid area distribution; **(C)** Representative IF staining showed that less E-cad and Snail expression with addition of LOX inhibitor (scale bars = 100 μm); **(D)** Semi-quantitative measurement of fold changes of cell viability, spheroid density, positive E-cad and Snail cell density comparing between with and without LOX inhibitor; **(E)** Representative IF staining for vimentin (red), E-cad (green) and nuclei (blue) for migrated 21PT and LMC with LOX inhibitor after 14-day culture (scale bars = 100 μm); **(F)** Semi-quantitative measurement of fold changes of migrated 21PT density with LOX inhibitor comparing to with and without LMC without LOX inhibitor (n=5; # indicates significant decrease of migrated 21PT comparing to hypoxia LMC- condition; $ indicates significant decrease of migrated 21PT comparing to hypoxia LMC+ condition; ^*^ indicates significant increase of migrated 21PT comparing to hypoxia LMC- with LOX inhibitor; p<0.05).

Since 21MT-2 had limited differences in LOX and LOX like family expression in response to hypoxic conditioning, we focused on studying the effects of LOX inhibitor (i.e., BAPN) on 21PT cell line migration. Figure 6A and 6B showed that with addition of BAPN most 21PT were still alive, and self-assembled with larger spheroid size and broader distribution compared to their counterparts without LOX inhibitor. The LOX inhibitor did not affect 21PT cell viability, but significantly increased spheroid density (Figure 6D). In addition, 21PT expressed E-cadherin and Snail (Figure 6C), expression of both of these proteins decreased by the addition of LOX inhibitor (Figure 6D). The addition of LOX inhibitor also decreased 21PT migration capacity to hydrogels with and without populated LMC (Figure 6E, 6F). The migrated E-cadherin positive cell density in LMC laden constructs decreased around 2-fold in comparison with the group without LOX inhibitor treatment (Figure 6E).

## DISCUSSION

Three dimensional breast cancer cell models are becoming increasingly popular to recreate a cancer-like microenvironment for better understanding of cancer cell behavior, identifying potential targets for cancer treatment, and screening anticancer drugs [44, 45]. In this study, we implemented HA/Gel based hydrogels to encapsulate breast cancer cells as a 3D model to investigate the effects of hypoxia on cancer cell behavior. HA is found associated with the connective stroma and has increased level in breast tumor tissue in comparison with the non-tumor counterpart [46]. Gelatin is denatured collagen, which is major breast tumor ECM component [47]. The modified Me-HA/Me-Gel hydrogels have tunable stiffness [48, 49], and have the capacity to incorporate various growth factors [50]. We implemented the hydrogel recipe that provided the stiffness comparable to the native adipose tissue.

Various breast cancer cell lines from different subtypes, like MCF-7 (luminal A), BT474 (luminal B), SKBR3 (human epidermal growth factor receptor 2 -HER2), MDA-MB-231(triple negative), have been used in 3D microenvironment. In this study, we used isogenic primary 21PT and metastatic 21MT-2 cell lines, which were derived from the same HER2 positive patient at different tumor stages [51–53]. 21PT are noninvasive cell line and represent infiltrating ductal carcinoma with carcinoma in situ component, which represent early stage of breast cancer, whereas 21MT-2 is a metastatic cell line and is derived from pleural effusion of lung metastasis [54, 55].

Hypoxia is known to promote breast cancer cell EMT [56], migration [57], and metastasis [31], via HIF-1 pathway [58] and other signaling pathway like unfolded protein response [59]. Our results demonstrated that hypoxia delayed or partially inhibited breast cancer cell spheroid formation within our hydrogel system. This probably is because hypoxia induced Snail expression facilitated EMT process. Lundgren et al also demonstrated that hypoxia induces EMT, but generally not a migratory phenotype even though levels of mesenchymal markers such as vimentin were increased and E-cadherin levels decreased [60]. Our hydrogel migration assays showed that 21PT released from 3D culture had much higher migration capability under hypoxic condition in comparison to normoxia, whereas 21MT-2 cells did not exhibit significant differences under the same conditions. This indicates that the migration capacity in response to hypoxic treatment is probably dependent on stage of the cancer. In addition, hypoxia promoted more LOX and LOX like enzymes expression for 21PT in comparison with 21MT-2 cell line. Again, this indicates that LOX (and LOX like enzymes) secretion and expression in response to hypoxia is stage dependent. Such stage dependent response of 21T series of breast cancer cells to hypoxia may be due to their original microenvironment. In the early stage, the breast cancer cells are subjected to intratumoral hypoxia, whereas metastatic breast cancer cells actually thrive in normoxic environment. This probably can also explain why 21MT-2 showed more EMT related gene expression in hypoxia and after exposure to normoxia the released 21MT-2 had comparable EMT related gene expression in comparison to without any hypoxic pre-treatment.

The communication between disseminated breast cancer cells and resident cells in different tissues is diverse and mostly unknown. Erler et al. reported that LOX secreted by hypoxic breast tumor cells accumulated at pre-metastatic sites and significantly affected bone metastasis [39]. Global quantitative analysis of the hypoxic secretome identified LOX as significantly associated with bone-tropism and relapse [37]. However, much less is known about the effects of hypoxia induced LOX on breast cancer lung metastases. In the current study, we developed the connection of isogenic LMC model with 3D breast cancer cell laden hydrogels and LMC laden hydrogels. Our results demonstrate that both LMC and hypoxia were crucial for facilitating 21PT migration, and hypoxia may be even more important. This indicates that the secretomes from 21PT laden constructs with hypoxia pre-treatment can mediate the gelatin hydrogel surface remodeling for 21PT further migration even without the stromal cells. Gilkes et al. demonstrated that HIF-1 activated the transcription of genes encoding collagen prolyl 4-hydroxylase (P4H) which can catalyze collagen proline hydroxylation and promotes cancer cell alignment along collagen fibers, resulting in enhanced invasion and metastasis to lymph nodes and lungs [41]. This indicates that both procollagen secretion (P4H related) and further triple-helical molecules crosslinking (LOX related) are important regulatory processes for breast cancer lung metastasis. Our results showed that the use of LOX inhibitor, i.e. BAPN, partially counteracted the effects of hypoxia by promoting cell assembly, decreasing EMT, and inhibiting 21PT migration to LMC laden constructs without affecting cell viability. Similarly, Bondareva et al. also demonstrated that the administration of BAPN prevented MDA-MB-231 cells metastases, but had no effect on the growth of established metastases [61]. LOX inhibition probably affects both normal cells and cancer cells by decreasing ECM deposition and maturation. Therefore, LOX and LOX family members may not be good therapeutic targets. However, LOX expression and activity are associated with hypoxia and may be a potential endpoint target to screen the pharmacological drugs. Taken together, our results demonstrate that the hydrogels based models can be utilized to study breast cancer cell behavior in different stages of tumor progression, as well as for assessing the therapeutic efficacies of hypoxia pathway inhibitors for the development of novel therapeutic agents against breast cancers.

## MATERIALS AND METHODS

### Cell culture

Culture conditions of 21PT and 21MT-2 breast cancer cell lines as well as LMC line have been described previously [52, 55].

### Polymer modification, hydrogel preparation and cell encapsulation

Photocrosslinkable Me-HA (HA, ∼1200 kDa, NovaMatrix) and Me-Gel (type B gelatin, Sigma) were synthesized as previously reported [62] through the reaction of methacrylic anhydride (Sigma) with HA (0.5%) and gelatin (10%) solution. A hydrogel precursor solution composed with Me-HA (0.75%w/v) /Me-Gel (0.75%w/v) was dissolved in cell culture medium with 0.05% w/v 2-hydroxy-1(4-(hydroxyethyl)phenyl)-2-methyl-1-propanone (Irgacure 2959, CIBA Chemicals). The gel precursor was transferred into silicone molds (8 mm in diameter, 1mm in thickness) and subsequently exposed to OmniCure S2000 UV lamp (Lumen Dynamics) for 45 s at room temperature to generate hydrogel matrix. For breast cancer cell encapsulations into hydrogels, 21PT or 21MT-2 cell lines were used at a density of 5×10^6^ cells/ml (Figure 1A). These cell-laden hydrogels scaffolds were maintained in the cell culture medium at 37°C in either a normoxic (5% CO_2_, 21% O_2_) or hypoxic (5% CO_2_, 5% O_2_) environment for 14 days in trigas incubator (Thermo Fisher Scientific). The medium was replaced on alternate days.

### Physical characterization of the hydrogel scaffolds

Surface morphologies of Me-HA/Me-Gel hydrogel scaffolds were characterized by utilizing SEM (FEI Quanta 200) after freeze-drying (Supplementary Figure 1). The stiffness of hydrogel scaffolds was conducted using a compression tester (Nanosute, Agilent Techologies) at room temperature.

### Cell viability and morphology

The viability of encapsulated cells within the hydrogels was determined after 14-day culture by using Live/Dead assay (Thermo Fisher Scientific) as previously described [63] and fluorescence images were obtained using a confocal laser scanning microscopy (CLSM, LSM 710, Carl Zeiss, Germany).

### Analysis of self-assembled breast cancer cell spheroid density and area distribution

Based on Live/Dead assay images for encapsulated 21PT or 21MT-2 cells within hydrogels, we calculated the self-assembled cellular spheroid density and area distribution in hydrogels in different oxygen tension environment. Spheroid density was quantified by counting the spheroid number via ImageJ and standardizing to image area. NIH ImageJ was used to measure the spheroid area and then the histograms were plotted. Three samples for each condition were used and three images from each sample were analyzed.

### IF staining

All samples were fixed in 4% paraformaldehyde, permeabilized in 0.2% Trion X-100 and then blocked with 1% bovine serum albumin (BSA) overnight at 4°C. The samples were then treated with primary antibodies to E-cadherin (E-cad, 1:100, Abcam), Snail (1:100, Abcam), vimentin (1:100, Sigma) overnight at 4°C. Secondary fluorescent antibodies were incubated for 2 h and nuclear counterstaining (Draq 5, 1: 1000, Thermo Fisher Scientific) were performed for 30 minutes at room temperature. The stained samples were imaged with Zeiss 710 CLSM. Based on the images, the density of E-cad positive and Snail positive cell density within the hydrogels were calculated by splitting the image channel and counting specific marker positive cell number via ImageJ and standardizing to image area. Three samples for each conditions were used and at least three images from each sample were analyzed.

### RNA isolation and qPCR

Total RNA was extracted from cell-encapsulated hydrogel constructs or cell spheroids cultured in hydrogel-free medium using QIA-Shredder and RNeasy mini-kits (QIAgen) according to the manufactures’ instructions. Total RNA was synthesized into first strand cDNA in a 20 μL reaction using iScript cDNA synthesis kit (BioRad Laboratories). Real-time PCR analysis was performed in a StepOnePlus™ Real-Time PCR System (Thermo Scientific) using SsoAdvanced SYBR Green Supermix (Bio-Rad). cDNA samples were analyzed for the gene of interest and for the housekeeping gene 18S rRNA. The level of expression of each target gene was calculated using comparative Ct (2^–ΔΔCt^) method. All primers used in this study are listed in Supplementary Table 1.

### Western blot

Cell lysates were collected from 21PT cells cultured in 2D and 3D. Western blot was performed with indicated antibodies E-cad, (ab76055), Twist (ab50581), β-actin (ab 8227) from abcam, vimentin (clone SP20, RM-9120-S0) was from Thermo Scientific; Snail (LS- C176686) from LS Bio.

### LOX activity assay

The media for 21PT or 21MT-2 laden hydrogels were collected at the determined time points and frozen at -80°C until further use. LOX activity was measured in the media using a LOX activity assay (Abcam) according to the manufacturer’s instruction.

### Breast cancer cell migration assay

Two models were developed to evaluate breast cancer cell migration and metastasis capacity. In the first model, as shown in Figure 4A, the 3D cell laden hydrogel constructs were conditioned in either normoxic or hypoxic environment for 14 days and then were smashed to release the encapsulated cells. The released cells were reseeded onto the 2D 6-well culture plates for another 3 days in normoxia and then collected for either qPCR assay or transwell/Boyden chamber assay (Corning Costar). An MTT assay was used to determine the migrated cell metabolism in the bottom chamber receiver plate. In the second model, as shown in Figure 5A, LMC were seeded onto Me-HA/Me-Gel hydrogel disc (0.75%Me-HA and 6% Me-Gel with diameter of 15 mm for 60s photocrosslink, 2×10^4^ cells/disc) and conditioned in normoxia for 14 days. During the same time, 21PT laden hydrogel constructs were cultured in either normoxia or hypoxia for 14 days. Then the 21PT laden hydrogel constructs were stacked onto the Me-Gel hydrogel discs with or without LMC with a transwell chamber (the bottom porous membranes were removed) on the top to make sure the hydrogel discs were contacted and then subjected to LMC medium in normoxic environment for another 14 days, and then the top hydrogels with cells were removed and the bottom Me-Gel hydrogels were fixed for IF staining. The migrated cell density was calculated by counting the E-cadherin positive cell number via ImageJ and standardizing to image area. Three samples for each condition were used, and at least three images from each sample were analyzed.

### LOX inhibitor treatment

The 21PT laden hydrogels were treated with the LOX inhibitor beta-aminopropionitrile (BAPN, 500μM, Sigma) during 14-day culture in normoxia or hypoxia conditions. Then the Live/Dead assay, qPCR and IF staining were conducted using the method described previously [64, 65]. The migration capacity of 21PT cells to the LMC laden hydrogel and through the transwell chamber after smashing the hydrogels was evaluated after BAPN treatment, using the method described in Section 2. 9.

### Statistical analysis

All quantitative data is shown as mean ± standard deviation (SD). Pairwise comparisons between groups was performed using ANOVA with Scheffé post-hoc tests in statistical analysis. A value of p < 0.05 was considered statistically significant.

## SUPPLEMENTARY MATERIALS FIGURE AND TABLE



## Abbreviations

ECM: Extracellular matrix
EMT: Epithelial to mesenchymal transition
LOX: Lysyl oxidase
LMC: Lung mesenchymal cells
HIF: Hypoxia-inducible factors
Me-HA: Methacrylated hyaluronic acid
Me-Gel: Methacrylated gelatin
SEM: Scanning electron microscopy
