# Supplementary material for: 3D hydrogel breast cancer models for studying the effects of hypoxia on epithelial to mesenchymal transition
Source: Oncotarget. 2018 Aug 14;9(63):32191–203. doi: 10.18632/oncotarget.25891 (PMC6114943; doi:10.18632/oncotarget.25891)
Supplement: Supplementary file 1 [file oncotarget-09-32191-s001.pdf]

3D hydrogel breast cancer models for studying the effects of hypoxia on epithelial to mesenchymal transition

SUPPLEMENTARY MATERIALS

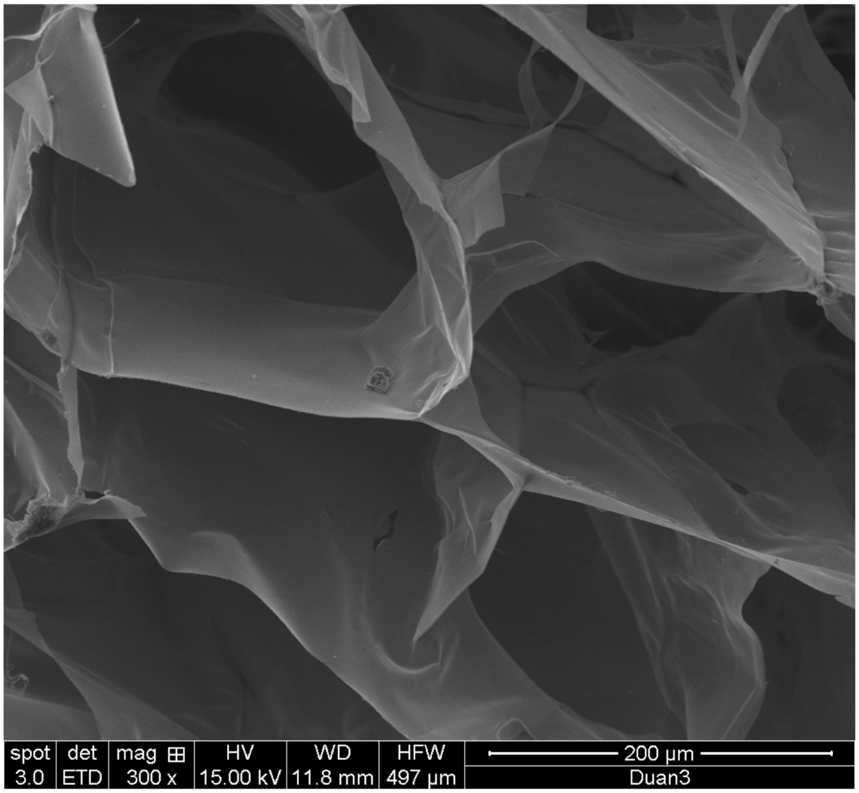

Supplementary Figure 1: Scanning electron microscopic image of Me-HA/Me-Gel hydrogel.

Supplemental Table 1: qPCR Primers

Gene symbol	Genbank ID	Primer sequences (5'→3')	Product size (bp)
ACTB	NM_001101.4	F: GAAGTGTGACGTGGACATCC<br>R: CCGATCCACACGGAGTACTT	177
CDH1	NM_001317184.1	F: CGAGAGCTACACGTTCACGG<br>R: TTTGAATCGGGTGTCGAGGG	127
CDH2	NM_001308176.1	F: GGGAAATGGAAACTTGATGGCA<br>R: GCAGGCTCACTGCTCTCATA	158
SNAI-1	NM_005985.3	F: CTAGGCCCTGGCTGCTACAA<br>R: GACATCTGAGTGGGTCTGGAG	131
VEGFA	NM_001025366.2	F: CTGGAGCGTGTACGTTGGT<br>R: TGCAACGCGAGTCTGTGTTT	155
MMP-1	NM_002421.3	F: GACAGCCTCTGGCTTTCTGG<br>R: AGTGGAGGAAAGCTGTGCAT	152
HIF1A	NM_001530.3	F: ATCACCCTCTTCGTCGCTTC<br>R: GGAAAGGCAAGTCCAGAGGT	164
LOX	NM_002317.6	F: CTTGCACGTTTCCAATCGCA<br>R: GGCAGTGTCTGGAGTGAAGG	130
LOXL1	NM_002317.6	F: CTTGCACGTTTCCAATCGCA<br>R: GGCAGTGTCTGGAGTGAAGG	130
LOXL2	NM_002318.2	F: CCAGTGTGGTCTGCAGAGAG<br>R: CCTGTGCACTGGATCTCGTT	112
LOXL3	NM_032603.4	F: CAGCCCTCCCACCTCATTTT<br>R: TCCTTGCCTTTCTGGCTGAG	184
LOXL4	NM_032211.6	F: GAGGCGGATAAAAAGGCCCC<br>R: GGCCAAGATACCAGATGGAGC	110
